# Genomic Diversity of *Listeria monocytogenes* Isolates From Slovakia (2010 to 2020)

**DOI:** 10.3389/fmicb.2021.729050

**Published:** 2021-11-02

**Authors:** Zuzana Kubicová, Sophie Roussel, Benjamin Félix, Lenka Cabanová

**Affiliations:** ^1^State Veterinary and Food Institute (SVFI), Dolny Kubin, Slovakia; ^2^Maisons-Alfort Laboratory for Food Safety, Salmonella and Listeria Unit, University of Paris-Est, French Agency for Food, Environmental and Occupational Health & Safety (ANSES), Maisons-Alfort, France

**Keywords:** *Listeria monocytogenes*, serotyping, PFGE, WGS, genomic diversity

## Abstract

Over the past 11 years, the Slovak National Reference Laboratory has collected a panel of 988 *Listeria monocytogenes* isolates in Slovakia, which were isolated from various food sectors (61%), food-processing environments (13.7%), animals with listeriosis symptoms (21.2%), and human cases (4.1%). We serotyped these isolates by agglutination method, which revealed the highest prevalence (61.1%) of serotype 1/2a and the lowest (4.7%) of serotype 1/2c, although these represented the majority of isolates from the meat sector. The distribution of CCs analyzed on 176 isolates demonstrated that CC11-ST451 (15.3%) was the most prevalent CC, particularly in food (14.8%) and animal isolates (17.5%). CC11-ST451, followed by CC7, CC14, and CC37, were the most prevalent CCs in the milk sector, and CC9 and CC8 in the meat sector. CC11-ST451 is probably widely distributed in Slovakia, mainly in the milk and dairy product sectors, posing a possible threat to public health. Potential persistence indication of CC9 was observed in one meat facility between 2014 and 2018, highlighting its general meat-related distribution and potential for persistence worldwide.

## Introduction

*Listeria monocytogenes* is a Gram-positive intracellular bacterium responsible for a serious food-borne zoonosis called listeriosis. This pathogen is transmissible to humans through the consumption of contaminated food. It can lead to mild gastroenteritis and also to serious infections of the bloodstream or the central nervous system as well as abortions. The organism is able to pass from cell to cell, allowing it to cross blood–brain and placental barriers (Janakiraman, 2008). Immuno-compromised persons, pregnant women, infants, and the elderly are the most vulnerable to listeriosis (Vazquez-Boland et al., 2001). In 2015–2019, the listeriosis trend in Europe remained stable after a long period of increase but had the highest case-fatality rate (8.9%) among the outbreak-related illness (EFSA and ECDC, 2021). In Slovakia, 18 human listeriosis cases and four deaths were reported in the year 2019, which is 6% above the 5-year average (Slovak Focal Point for the Scientific, and Technical Matters of the Efsa., 2020).

Some *L. monocytogenes* strains can persist for a long period of time in various types of food-processing environment (FPE), including chilled processing plants (Lundén et al., 2004; Schmitz-Esser et al., 2015). *Listeria monocytogenes* contamination may occur as a result of poor hygiene during food processing or packaging (Carpentier and Cerf, 2011).

Animal listeriosis is generally subclinical, but more serious forms have also been observed (OIE, 2018), with symptoms including septicaemia, encephalitis, meningitis, meningoencephalitis, rhombencephalitis, abortion, stillbirth, perinatal infections, and/or gastroenteritis (Okada et al., 2011; OIE, 2018).

*Listeria monocytogenes* is a genetically heterogeneous species divided into four phylogenetic lineages, of which lineages I and II are the most frequently encountered. The strains from lineage I (serotypes 1/2b, 4b) are in general found in higher frequency in human outbreaks than lineage II strains (serotypes 1/2a, 1/2c), which are commonly isolated from food, natural and farm environments, and animal and sporadic human listeriosis cases (Orsi et al., 2011).

Several methods have been developed to investigate the genetic diversity of *L. monocytogenes* strains. Agglutination serotyping is considered to be the first level of discrimination between isolates and can differentiate 13 serotypes. For many years, pulsed-field gel electrophoresis (PFGE) had been the gold standard for *L. monocytogenes* subtyping (Graves and Swaminathan, 2001). Another key typing approach has been multi-locus sequence typing (MLST) based on the standardized nomenclature derived from the sequences of seven housekeeping genes (Ragon et al., 2008). Unique combinations of alleles from MLST analysis determine strain sequence types (STs) and clonal complexes (CCs), which are now systematically used to describe the population structure of *L. monocytogenes* (Ragon et al., 2008; Cantinelli et al., 2013; Maury et al., 2016). Certain CCs account for the majority of outbreaks and sporadic cases in humans (Maury et al., 2016) and animals (Dreyer et al., 2016). More recently, typing based on whole-genome sequencing (WGS) of *L. monocytogenes* has become a very powerful tool and more and more studies are employing this method for national surveillance, outbreak detection, or tracking of the sources of listeriosis (Jackson et al., 2016; Moura et al., 2017; Pietzka et al., 2019).

The State Veterinary and Food Institute (SVFI) has been the designated Slovak National Reference Laboratory (NRL) for *L. monocytogenes* since 2007. This national-level laboratory is engaged in the surveillance and typing of *L. monocytogenes* isolates from food items and animals. It participates in the European surveillance network for *L. monocytogenes*, along with 34 other NRLs coordinated by the European Reference Laboratory (EURL).^1^ The PFGE method is routinely performed in the Slovak NRL. The isolates are assigned to an MLST CC using a mapping method detailed in Félix et al. (2018). Whole-genome sequencing of the isolates is currently being introduced.

Only a few studies are available on the genomic or genetic diversity of *L. monocytogenes* in Slovakia, and they focus only on selected food manufacturers, either in the dairy sector (Véghová et al., 2015) or in the meat sector (Véghová et al., 2016). To date, no data are available on a large and diverse panel of food isolates for Slovakia. The purpose of this study was thus to provide an overview of the population structure of the *L. monocytogenes* isolates over the past 11 years in Slovakia. This survey should lead to better management and understanding of food-related health risks.

This study had two objectives. The first was to analyse the genetic diversity of all *L. monocytogenes* isolates from food, FPEs, and animals available in the NRL collection. The second was to compare this diversity to that of isolates from human outbreaks during the same time period. The genetic diversity of 988 isolates was assessed using conventional agglutination serotyping. In a subset of 176 isolates, CCs were deduced either from PFGE profiles (127 isolates) or from WGS analysis (49 isolates).

## Materials and Methods

### Isolate Panel

A panel of 988 isolates collected during the 2010–2020 period was selected for this study (Supplementary Table 1). The isolates were from four compartments: food, FPEs, animals, and humans.

The vast majority of the isolates (947) were from the NRL microbial collection: 603 (61%) isolates were from food, 135 (13.7%) from FPEs, and 209 (21.2%) from animals (40.7% of animal isolates were isolated from sheep; 29.2% from cattle) with clinical manifestations of listeriosis. Detailed epidemiological information (sampling stage, context, sources, food matrix, and food product) were compiled in the NRL molecular database (BioNumerics, vers. 7.6.3, Applied Maths, Sint-Martens-Latem, Belgium).

**FIGURE 1:**
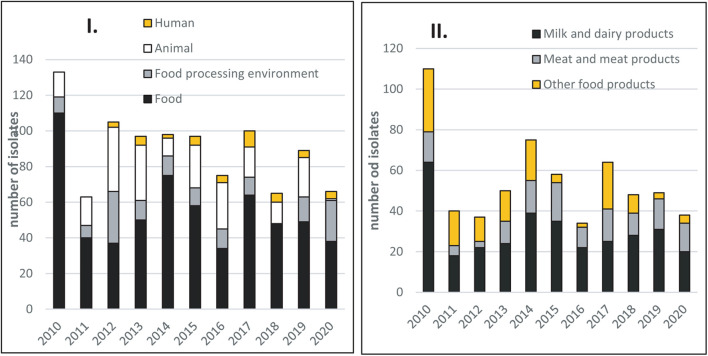
**(I)** Distribution of *Listeria monocytogenes* isolates collected yearly from food, food-processing environments, animals, and human cases of listeriosis between 2010 and 2020 in Slovakia. **(II)** Distribution of *Listeria monocytogenes* isolates according to food origin during the 2010–2020 period.

The number of food isolates per sampling period (2010–2020) was generally evenly distributed across years, with an average of 54.8 isolates per year (Figure 1). The food isolates included 328 (54.5%) isolates from milk and dairy products, 135 (22.4%) from meat and meat products, and 140 (23.1%) from other food products (ready-to-eat delicatessen products predominated, representing 85.6% of isolates in this category). Another 135 (13.7%) isolates were from FPEs, mainly from dairy facilities (Figure 1). The 603 food isolates were from official Slovak controls and from the producer’s own internal testing procedures. The 209 (21.2%) animal isolates were collected by private veterinarians.

These food, FPE, and animal isolates were supplemented with 41 isolates (4.1%) from human listeriosis cases (Figure 1). These human case isolates were collected between 2012 and 2020 by the Slovak Public Health Authority (PHA), and sent to the NRL.

A subset of 176 isolates was then selected from the available typing data collection. The majority of isolates were chosen from 2014 (*n* = 51; 29%), 2015 (*n* = 41; 23.3%), and 2018 (*n* = 29; 16.5%) years in which intensive typing had been performed in Slovakia during national surveillance programs. These 121 isolates were then supplemented with 55 isolates (31.2%) from the 2010–2020 period, with PFGE or WGS typing data available. Whereas origin of the isolates is very constant throughout time, we considered this selection to be representative extraction of the whole panel. This subset (*n* = 176) included isolates from four compartments in particular: 115 food sector isolates (65.3%), with 68 dairy isolates (59.2%), 28 meat isolates (24.3%), and 19 isolates (16.5%) from other foods. Of the 61 remaining isolates, 21.3% were from FPEs, 65.6% from animals, and 13.1% from human cases (Supplementary Table 1).

### *Listeria monocytogenes* Isolates Recovery

The isolates were recovered from a long-term storage at –80°C using tryptone soya yeast extract agar (TSYEA, Biokar Diagnostics, Allonne, France).

### Agglutination Serotyping (988 Isolates)

Serotypes can be identified based on the somatic (O) and flagellar (H) antigens. Antisera against eight somatic (OI/II, OI, OIV, OV/VI, OVI, OVII. OVIII, OIX) and four flagellar (HA, HAB, HC, HD) antigens were used as recommended by the manufacturer of a commercially distributed kit (Denka Seiken, Tokyo, Japan).

### Determination of Clonal Complexes and Sequences Types (176 Isolates)

#### Molecular Typing Using Pulsed-Field Gel Electrophoresis (127 Isolates)

Pulsed-field gel electrophoresis (PFGE) was performed according to the protocol described in Roussel et al. (2014) in the years of isolate collection. Electrophoresis was performed on 1% agarose gel (SeaKem Gold Agarose, Lonza, Rockland, ME, United States) with a CHEF Mapper^®^ XA system (Bio-Rad, Hercules, CA, United States). *Salmonella* serovar Braenderup H9812 DNA digested by *Xba*I enzyme (Thermo Scientific, Vilnius, Lithuania) was used as a reference standard.

BioNumerics software (vers. 7.6.3) was used to create a database and compare the PFGE profiles of 164 *L. monocytogenes* isolates. Epidemiological duplicates were excluded. They were defined as isolates sharing indistinguishable PFGE profiles, isolated the same year and provided by the same food business operator or the same diagnostic food laboratory, as described in Félix et al. (2018). A total of 164 PFGE profiles were interpreted according to the standard operating procedure (SOP) detailed in Michelon et al. (2015). One hundred thirty-four isolates were grouped to clusters according to the 85% similarity rule. Of these, five *L. monocytogenes* isolates were selected for WGS, through the H2020 European Joint Program (EJP) ListAdapt (Félix et al., 2020), and two human ST451 isolates (deduced from PFGE) were sequenced at the Slovak NRL, resulting in 127 PFGE profiles (Supplementary Figure 1 dendrogram) for mapping to MLST data. For these 127 isolates, the CCs were deduced from the PFGE profiles, using the mapping protocol detailed in Félix et al. (2018).

#### Whole-Genome Sequencing and Multi-Locus Sequence Typing (49 Isolates)

A total of 49 isolates were whole-genome sequenced: 45 isolates were sequenced by external partners as part of European research projects, such as the H2020 European Joint Program (EJP) ListAdapt (Félix et al., 2020) and the EFSA-LISEQ project (Nielsen et al., 2017; Painset et al., 2019); 1 isolate was sequenced by the Austrian NRL; and 3 isolates were sequenced by the Slovak NRL in an iSeq 100 sequencing machine (Illumina). The 49 WGS datasets were assembled using SPADES and analyzed to determine their MLST (7 loci, Salcedo et al., 2003; Ragon et al., 2008), core-genome MLST (cgMLST) (1,748 loci, Moura et al., 2016), and whole-genome MLST (wgMLST) with 4,804 loci in BioNumerics software version 7.6.3 with connection to the EURL for the *Listeria monocytogenes* calculation engine. Raw sequencing reads were quality-checked according to the following criteria: PHRED score of raw reads greater than 30 and average depth coverage greater than 30 × (Neuman et al., 2013). The target length of the *de novo* assembly was between 2.8 and 3.1 Mb, and identification of 95%–100% of cgMLST loci was necessary for assignment to a CC-ST profile (Moura et al., 2016).

### Comparison of the Genomes of Seven ST451 Isolates With Publicly Available Genomes

Out of the 49 isolates for which the whole genome was sequenced, seven CC11-ST451 isolates were collected in Slovakia (four animal, one milk, and two human isolates). These seven genomes were compared with the 144 genomes of CC11-ST451 isolates available in public databases to see the possible relatedness between them as the CC11-ST451 was the most abundant in Slovak isolate collection and not typically present in Europe. One hundred six genomes were from NCBI-ENA databases, 8 genomes from the EFSA project LISEQ (Nielsen et al., 2017), 29 genomes from Lüth et al. (2021), and 1 genome from Kuch et al. (2018) (Supplementary Table 2). The genomes were quality-checked and compared using cgMLST (1748 loci, Moura et al., 2016) of all the ST451 isolates, as described in Section “Whole-Genome Sequencing and Multi-Locus Sequence Typing (49 Isolates).”

## Results

### Serotype Distribution According to the Compartment and Food Sector

Out of the 988 isolates studied, the majority (61.1%) belonged to serogroup 1/2a, 17.8% to 1/2b, 12.7% to 4b, and 4.7% to 1/2c. The remaining isolates (3.7%) were assigned to other serotypes. Serotype 1/2a represented 72.0% of the isolates from the milk and dairy product sector, 64.4% from meat and meat product sector, 38.6% of other food sectors, 57.0% from the FPE sector, 63.6% from animal samples, and finally 41.5% from human cases. In the food sectors, serotypes 1/2b and 4b were mainly detected in food matrices other than dairy and meat (35.7% and 22.1%, respectively) (Table 1). The distribution of serotypes throughout the years 2010-2020 was mostly uniform with the average 62.5% representing 1/2a serotype per year (46.7%–75.3%), 16.9% of 1/2b serotype per year (6.2%–32.3%), 4.5% of 1/2c serotype per year (1.0%–9.2%), and 12.4% of 4b serotype per year (3.1%–32.4%).

**TABLE 1:** Distribution of *Listeria monocytogenes* serotypes according to isolate compartment and food sector.

Serotype (% of all serotyped isolates)	Number of isolates					
	Milk and dairy products (% of the individual serotypes)	Meat and meat products (% of the individual serotypes)	Other foods (% of the individual serotypes)	Food-processing environments (% of the individual serotypes)	Animal samples (% of the individual serotypes)	Human cases (% of the individual serotypes)
1/2a (61.1)	236 (72.0)	87 (64.4)	54 (38.6)	77 (57.0)	133 (63.6)	17 (41.5)
1/2b (17.8)	47 (14.3)	17 (12.6)	50 (35.7)	24 (17.8)	28 (13.4)	10 (24.4)
1/2c (4.7)	10 (3.1)	22 (16.3)	3 (2.2)	7 (5.2)	4 (1.9)	0 (0.0)
4b (12.7)	26 (7.9)	6 (4.4)	31 (22.1)	26 (19.3)	23 (11.1)	13 (31.7)
Other serotypes (3.7)	9 (2.7)	3 (2.3)	2 (1.4)	1 (0.7)	21 (10.0)	1 (2.4)

### Clonal Complexes Distribution According to the Compartment and Food Sector

**FIGURE 2:**
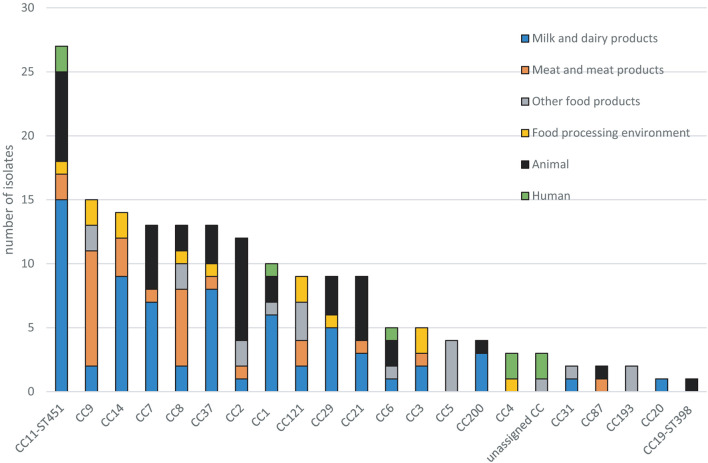
Distribution of clonal complexes (CCs) in the dataset of 176 *L. monocytogenes* isolates.

CC11-ST451 (serotype 1/2a) was the most prevalent CC in the panel of 176 isolates (15.3%, *n* = 27) (Figure 2 and Table 2). The origin of these CC11-ST451 isolates is summarized in Supplementary Table 1. In particular, the highest frequency of CC11-ST451 (22.1%, *n* = 15) was observed in dairy products (Figure 2 and Table 2).

**TABLE 2:** Distribution of clonal complexes (CCs) in the dataset of 176 *L. monocytogenes* isolates.

CC	Milk and dairy products	Meat and meat products	Other foods	Food processing environments	Animal samples	Human cases	Total
CC11-ST451	15	2	0	1	7	2	27
CC9	2	9	2	2	0	0	15
CC14	9	3	0	2	0	0	14
CC7	7	1	0	0	5	0	13
CC8	2	6	2	1	2	0	13
CC37	8	1	0	1	3	0	13
CC2	1	1	2	0	8	0	12
CC1	6	0	1	0	2	1	10
CC121	2	2	3	2	0	0	9
CC29	5	0	0	1	3	0	9
CC21	3	1	0	0	5	0	9
CC6	1	0	1	0	2	1	5
CC3	2	1	0	2	0	0	5
CC5	0	0	4	0	0	0	4
CC200	3	0	0	0	1	0	4
CC4	0	0	0	1	0	2	3
Unassigned CC	0	0	1	0	0	2	3
CC31	1	0	1	0	0	0	2
CC87	0	1	0	0	1	0	2
CC193	0	0	2	0	0	0	2
CC20	1	0	0	0	0	0	1
CC19-ST398	0	0	0	0	1	0	1
Total	68	28	19	13	40	8	176

The CC11-ST451 isolates analyzed here were isolated from very diverse milk and dairy products collected in a geographic area of roughly 200 km in diameter (north-western central Slovakia) throughout the 2014–2020 period. Many CC11-ST451 animal isolates (from clinical sheep cases, sheep’s milk) were also from the same region and collected during this same time period. These results revealed a wide distribution of CC11-ST451 in the north-western area of central Slovakia.

**FIGURE 3:**
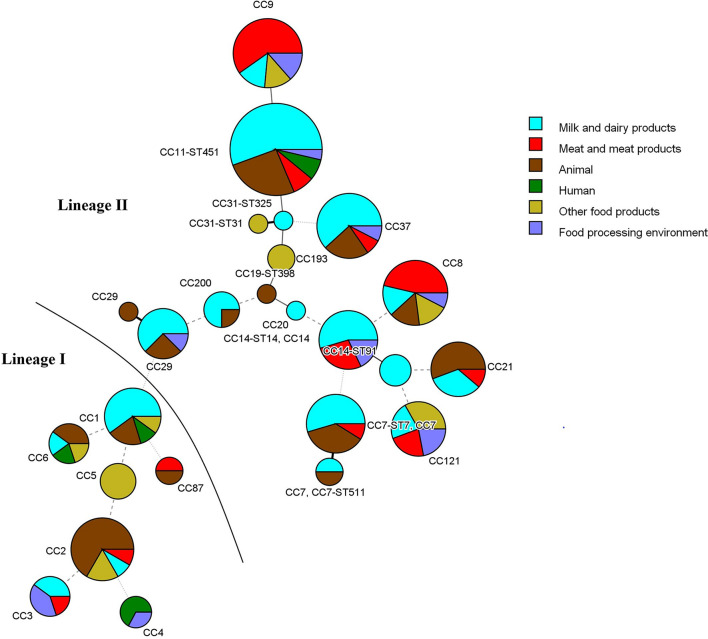
Minimum spanning tree of MLST data for the 173 *L. monocytogenes* isolates analyzed (3 isolates with unassigned CC were excluded). Each CC is represented by a circle whose size corresponds to the number of isolates in the clonal group. STs are known only for isolates that were whole-genome sequenced. Lines connecting the circles indicate phylogenetic relatedness and the number of allelic differences (thick solid line—1; thin solid line—2; dashed line—3; dotted line—4 and above).

The other prevalent CCs among dairy products and animals were CC7 and CC37 (Figure 2 and Table 2). Meat-related isolates of *L. monocytogenes* mainly grouped into CC9, followed by CC8. Human isolates belonged to CC11-ST451 (Lineage II), CC4, CC6, and CC1 (Lineage I) (Figure 2 and Table 2). The minimum spanning tree (Figure 3) shows the phylogenetic relatedness between the CCs.

### Indication of Potential Persistence in One Meat Product Facility

**FIGURE 4:**
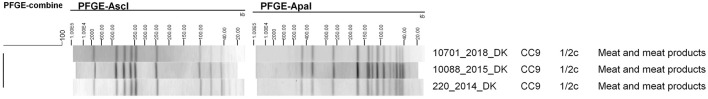
PFGE profiles obtained from *L. monocytogenes* isolates from one meat associated facility between 2014 and 2018.

The same PFGE profile was observed for three isolates collected between 2014 and 2018 in one meat product facility (Figure 4). This profile mapped to CC9 (serotype 1/2c).

### Comparison of the Genomes of Seven CC11-ST451 Isolates With Publicly Available Genomes

**FIGURE 5:**
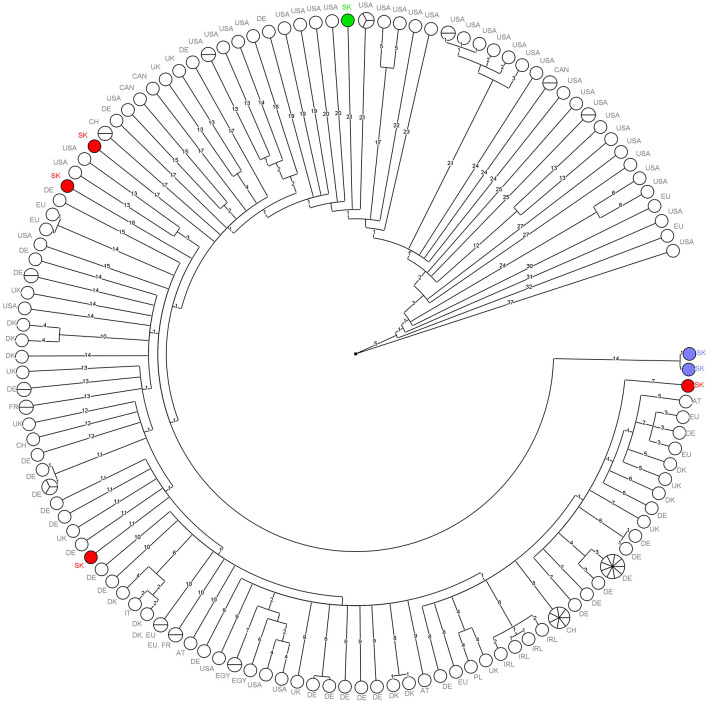
Minimum spanning tree of cgMLST data for 151 *L. monocytogenes* CC11-ST451 isolates. Branch numbers indicate the number of allelic differences between the isolates. Colored circles represent the seven Slovak ST451 isolates.

Out of the 27 CC11-ST451 isolates, 7 isolates were selected for WGS and compared using cgMLST, which showed at least 18 allele differences (AD) between each other, with the exception of human isolates that differed by only one AD. Subsequently, the seven isolates were compared with the CC11-ST451 isolates from public databases and external collaboration (Section “Comparison of the Genomes of Seven ST451 Isolates With Publicly Available Genomes”). None of the seven Slovak isolates showed fewer than seven AD with any other isolate (Figure 5). Moreover, the results of our work undertaken in collaboration with Austrian colleagues [Dr. Adriana Cabal, Austrian Agency for Health and Food Safety (AGES)] showed that the seven Slovak isolate genomes did not match with fewer than seven AD across the approximately 600 CC11-ST451 isolates present in the Austrian national database. The sequences of human isolates did not match any other isolate genomes but clustered together with only one AD in the cgMLST network.

## Discussion

Listeriosis is a serious public health and food safety problem in European countries, including Slovakia. Little data are available on the population genetic structure of food isolates in this country. Considering that food products are the main sources of human infections, it is very important to study the overall distribution of isolates throughout the country to improve understanding of the circulation of this disease.

The 988 *L. monocytogenes* isolates analyzed in this study by Denka Seiken kit which is often considered the “gold standard” for its serotyping (Burall et al., 2011) were isolated throughout the 2010–2020 period, providing a representative coverage of isolates circulating in Slovakia. Out of the 988 isolates tested using conventional serotyping, more than half (61.1%) were serotype 1/2a. This prevalence is consistent with many previous studies performed on isolates from various types of foods, mostly milk and dairy and meat products (Kramarenko et al., 2013; Martín et al., 2014; Ebner et al., 2015). The distribution of serotypes throughout the years suggested an even distribution of serotypes 1/2a, 1/2c, and 4b. The descending trend in distribution of 1/2b was observed in the 2010–2020 period.

In this study, CC1, CC2, CC4, and CC6 made up 66.7% of lineage I isolates and were isolated from food, FPEs, and animals, as well as from human cases. This result is similar to that reported in Lachtara et al. (2021) on the prevalence of CC1, CC2, and CC6 in food and FPE isolates in Poland. These CCs have previously been described as hypervirulent (Maury et al., 2016). CC1 and CC2 were concluded as being predominant in all world regions with an exception of northern Africa for CC1. Interestingly, CC6 had been observed mainly in Europe in a study of Chenal-Francisque et al. (2011).

Regarding the food isolates, serotypes 1/2b and 4b were detected mainly among the isolates from milk and dairy products and other food products, this latter category being predominated by ready-to-eat delicatessen products (mainly ready-to-eat salads). These isolates were collected mainly from production plants. Considering that the typing data in this category were only available for a limited number of isolates mainly from 2014, more extensive evaluation of the CC’s diversity should be performed in future to clarify its importance in public health protection. The decreasing trend of the 1/2b serotype in the period of 2010–2020 might be explained by the reduced popularity of rather unhealthy delicatessen products and increased healthier eating habits of Slovak population (Holotová et al., 2021). Prevalence of serotypes 1/2b and 4b has been reported in several studies on milk, meat, and ready-to eat products (Beak et al., 2000; Braga et al., 2017).

Serotype 1/2c was prevalent in isolates from meat, corroborating results from other studies conducted on the meat sector in Europe (Gianfranceschi et al., 2009; Ortiz et al., 2010; Prencipe et al., 2012; Meloni et al., 2014; Ebner et al., 2015; Morganti et al., 2015; Félix et al., 2018).

An indication of potential persistence of a CC9 *L. monocytogenes* isolate was observed at one meat production site between 2014 and 2018. This result substantiates previous studies demonstrating persistence of CC9 over many years in meat-processing facilities in Switzerland (Stoller et al., 2019) and in meat products and in pig slaughterhouse environments worldwide (Martín et al., 2014; Wang et al., 2015; Véghová et al., 2016; Félix et al., 2018; Maury et al., 2019; Demaître et al., 2020; Fagerlund et al., 2020).

In this study, two CCs, CC1 and CC37, were frequently observed among the isolates sourced from milk and dairy products and from sheep. This result is in accordance with a 2019 study (Papić et al., 2019) in Slovenia that showed that CC1 and CC37 were responsible for the majority of the clinical cases in animals, mainly small ruminants and cattle. CC37 is also the most prevalent isolate found on pig and ruminant farms in France (Félix et al., 2018) and frequently reported from ruminant farms in Switzerland (Dreyer et al., 2016), in the natural environment throughout Europe (Haase et al., 2014) and in the natural environment in Austria (Linke et al., 2014). Similar to our findings, CC7 and CC37 were the most abundant in the study focusing on milk and milk-related environment in the United States (Kim et al., 2018).

Interestingly, CC121 isolates, which are overrepresented in all food sectors in Europe and worldwide (Chenal-Francisque et al., 2011; Henri et al., 2016; Félix et al., 2018) and which can persist in FPEs (Holch et al., 2013; Rychli et al., 2017; Stoller et al., 2019), were of minor occurrence in our study, with only a few CC121 isolates from food sectors being observed. Also, CC121 belongs to the most prevalent clones worldwide (Chenal-Francisque et al., 2011). No CC121 isolates were isolated from animals, similar to previous reports (Linke et al., 2014; Dreyer et al., 2016; Félix et al., 2018).

In this study, CC11-ST451 was prevalent in the milk and dairy products sector. This result is concordant with an Austrian study that revealed that (i) dairy products are three times more likely to be contaminated with CC11-ST451 than the products from other food categories and (ii) a large part of CC11-ST451 food isolates could be traced back to a specific cheese-producing facility (Cabal et al., 2019). However, of the three ST451 human cases reported in Austria in 2017, none were related to the cheese-producing facility and all three were distinguishable, thus most likely arising from different contamination sources. CC11-ST451 isolates have been also reported in dairy products in the Czech Republic (Tomaštíková et al., 2019).

These findings, together with the frequent detection of CC7 in dairy products in Austria (Cabal et al., 2019) and the location of Austria and Czech Republic in Central Europe, are in accordance with our observation of CC11-ST451 and CC7 in the isolates from the dairy sector and the hypothesis of a multiple sources of introduction of these clones that likely circulate intensively.

CC11-ST451 isolates were also detected in the meat production sector, particularly in one rabbit meat-processing plant in Czech Republic. Persistence of these isolates in FPEs was first suggested by Gelbíčová et al. (2019). Interestingly, only one CC11-ST451 isolate was detected among the FPE isolates in our study.

Human cases of listeriosis caused by CC11-ST451 have been reported in Germany (Halbedel et al., 2018), France (Moura et al., 2017), Austria (Cabal et al., 2019), and Poland (Kuch et al., 2018). A nosocomial outbreak linked to ST451 was described lately in Italy (Russini et al., 2021). Furthermore, this clone is considered hypervirulent based on its prevalence in human cases and in food contamination in France (Fritsch et al., 2018).

Conversely, a very low occurrence of ST451 (fewer than 10 isolates) was reported in a wide-scale study (Painset et al., 2019) on 1,142 European isolates (isolated in 22 EU Member States and 1 Non-Member State). The frequent occurrence of CC11-ST451 in Slovakia and in neighboring countries suggests that this ST is specific to Central Europe.

The isolate distribution in Central Europe over a period of 11 years highlights a possible distribution of the CC11-ST451 clone in the milk and meat sectors in Slovakia. The seven genomes of Slovak CC11-ST451 isolates were dispersed among the available known genomes of ST451 isolates collected worldwide. The source of the CC11-ST451 isolates in Slovakia can potentially be determined with the systematic sequencing of the ST451 isolate genomes, now being collected by the Slovak NRL.

The importance of subtyping of *L. monocytogenes* isolates in Slovakia and in Europe in general is an important tool for food safety management. With the increasing production of food, globalization of the market, and consumers’ focus on traditional farm products with the characteristic small production and less trained staff in the field of the food safety, there is a higher risk of contamination of FPE and final food products. Isolate typing allows the identification of such threats and control measures implementation.

## Conclusion

This study is the first comprehensive insight into *L. monocytogenes* isolate diversity in Slovakia, covering isolates from the different food sectors available in the NRL collection, as well as isolates from clinical animal cases and several human outbreaks. The distribution of 988 serotypes was similar to that generally observed in Europe. The CC11-ST451 type, frequently reported in Central Europe, predominated in the Slovakia dataset, being detected mainly in isolates from dairy products.

## Data Availability Statement

The datasets presented in this study can be found in online repositories. The names of the repository/repositories and accession number(s) can be found below: http://www.ebi.ac.uk/ena/, PRJEB38828, PRJNA475189, and PRJEB45859.

## Author Contributions

LC was in charge of serotyping. ZK was in charge of PFGE data production, analysis, and interpretation. ZK and BF performed mapping of PFGE profiles to MLST CCs and WGS analysis. ZK, SR, and BF wrote the manuscript. All authors read, commented, and approved the final version.

## Conflict of Interest

The authors declare that the research was conducted in the absence of any commercial or financial relationships that could be construed as a potential conflict of interest.

## Publisher’s Note

All claims expressed in this article are solely those of the authors and do not necessarily represent those of their affiliated organizations, or those of the publisher, the editors and the reviewers. Any product that may be evaluated in this article, or claim that may be made by its manufacturer, is not guaranteed or endorsed by the publisher.

## Footnotes

1
https://eurl-listeria.anses.fr

## Supplementary Material

Supplementary Figure 1 Contains PFGE profiles for 127 strains which were mapped to MLST CC.
