# Supplementary figures and images for: Genomic Diversity of Listeria monocytogenes Isolates From Slovakia (2010 to 2020)
Source: Front Microbiol. 2021 Nov 2;12:729050. doi: 10.3389/fmicb.2021.729050 (PMC8593459; doi:10.3389/fmicb.2021.729050)

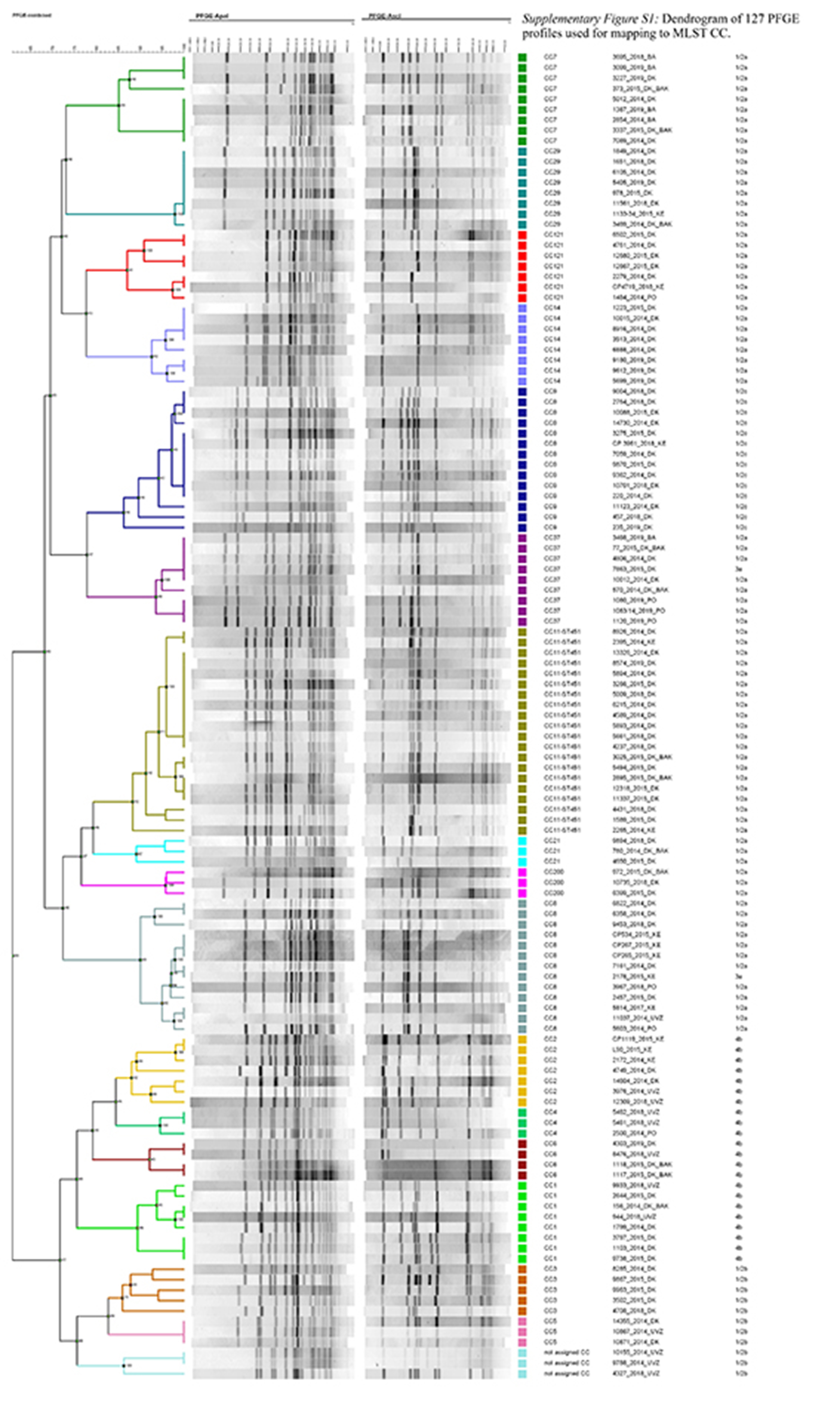

Supplement: Supplementary Figure 1 — Contains PFGE profiles for 127 strains which were mapped to MLST CC. [file Image_1.JPEG]
